# *Burkholderia cenocepacia* BC2L-C Is a Super Lectin with Dual Specificity and Proinflammatory Activity

**DOI:** 10.1371/journal.ppat.1002238

**Published:** 2011-09-01

**Authors:** Ondřej Šulák, Gianluca Cioci, Emilie Lameignère, Viviane Balloy, Adam Round, Irina Gutsche, Lenka Malinovská, Michel Chignard, Paul Kosma, Daniel F. Aubert, Cristina L. Marolda, Miguel A. Valvano, Michaela Wimmerová, Anne Imberty

**Affiliations:** 1 CERMAV-CNRS- UPR5301 affiliated to Université Joseph Fourier, Grenoble, France; 2 National Centre for Biomolecular Research and Department of Biochemistry, Faculty of Science, Masaryk University, Brno, Czech Republic; 3 European Synchrotron Radiation Facility, Grenoble, France; 4 Unité de Défense innée et Inflammation, Institut Pasteur, Paris, France; 5 INSERM U874, Paris, France; 6 EMBL, Grenoble, France; 7 UVHCI, UMI 3265 UJF-EMBL-CNRS, Grenoble, France; 8 Department of Chemistry, University of Natural Resources and Life Sciences, Vienna, Austria; 9 Centre for Human Immunology, Department of Microbiology and Immunology, University of Western Ontario, London, Canada; 10 Central European Institute of Technology, Masaryk University, Brno, Czech Republic; University of Michigan, United States of America

## Abstract

Lectins and adhesins are involved in bacterial adhesion to host tissues and mucus during early steps of infection. We report the characterization of BC2L-C, a soluble lectin from the opportunistic pathogen *Burkholderia cenocepacia*, which has two distinct domains with unique specificities and biological activities. The N-terminal domain is a novel TNF-α-like fucose-binding lectin, while the C-terminal part is similar to a superfamily of calcium-dependent bacterial lectins. The C-terminal domain displays specificity for mannose and l-*glycero*-d-*manno*-heptose. BC2L-C is therefore a superlectin that binds independently to mannose/heptose glycoconjugates and fucosylated human histo-blood group epitopes. The apo form of the C-terminal domain crystallized as a dimer, and calcium and mannose could be docked in the binding site. The whole lectin is hexameric and the overall structure, determined by electron microscopy and small angle X-ray scattering, reveals a flexible arrangement of three mannose/heptose-specific dimers flanked by two fucose-specific TNF-α-like trimers. We propose that BC2L-C binds to the bacterial surface in a mannose/heptose-dependent manner *via* the C-terminal domain. The TNF-α-like domain triggers IL-8 production in cultured airway epithelial cells in a carbohydrate-independent manner, and is therefore proposed to play a role in the dysregulated proinflammatory response observed in *B. cenocepacia* lung infections. The unique architecture of this newly recognized superlectin correlates with multiple functions including bacterial cell cross-linking, adhesion to human epithelia, and stimulation of inflammation.

## Author Summary

The glycoconjugates that cover the surface of eukaryotic cells are a target for pathogens that use protein receptors for binding to the carbohydrate moieties exposed. Opportunistic bacteria such as *Pseudomonas aeruginosa* and *Burkholderia* species of the *B. cepacia* complex display a wide range of adhesins and soluble lectins that are specific for human oligosaccharides. We characterized the complex architecture of one *Burkholderia cenocepacia* soluble lectin, and analysed the specificity of two different lectin subdomains. We propose that one of the subdomains attaches to sugars present on the bacteria surface, enabling bacterial aggregation in microcolonies. The other subdomain attaches to sugars in human airways. In addition, this domain can elicit an inflammatory response in airways cells. *Burkholderia cenocepacia* causes lethal infections in cystic fibrosis patients and soluble lectins may be novel therapeutics targets.

## Introduction

The *Burkholderia cepacia* complex (Bcc) is a group of Gram-negative bacteria comprising at least 17 species [1]. Bcc species are common in the environment and can be isolated from various sources including water, soil and vegetation. Bcc bacteria are involved in symbiosis and other interactions with plants that are beneficial for agriculture, but they are also recognised as important opportunistic human pathogens. In particular, *B. cenocepacia* causes infections in patients suffering from chronic granulomatous diseases [2] and cystic fibrosis [3] with significant morbidity and mortality. This is in part due to the extreme resistance of *B. cenocepacia* strains to almost all clinically useful antibiotics and their transmissibility between patients [4]. *B. cenocepacia* isolates survive either extracellularly in the airways or intracellularly within epithelial and phagocytic cells [5].

Among the virulence factors of *B. cenocepacia* [6], soluble lectins bind to carbohydrates present on epithelial cells and mucus [7]. A family of four soluble lectins has been identified in *B. cenocepacia*, all of them containing at least one domain with strong sequence similarity with LecB (PA-IIL) from *Pseudomonas aeruginosa*. LecB is a tetrameric fucose-binding lectin with unusually high affinity for carbohydrate mediated by two bridging calcium ions in its binding site [8]. Its structure has been elucidated and the involvement in biofilm formation and epithelial cell adhesion has been demonstrated [9], [10].

The four soluble lectins of *B. cenocepacia* are designated BC2L-A, -B, -C and –D. BC2L-A, consisting of one LecB like domain, associates as a dimer and binds mannose and oligomannose-type N-glycans [7], [11]. The three other lectins have additional N-terminal domains. The N-terminal domain of BC2L-C has been recently characterized as a novel fucose binding domain with a TNF-α-like fold [12]. Thus, BC2L-C encompasses two lectin domains probably assembling as oligomers, but nothing is known about its architecture and biological role.

Here, we characterize the structure and specificity of the C-terminal domain of BC2L-C. We also compare the specificity of the whole lectin with that of each domain. The overall hexameric architecture of the lectin was determined in solution by electron microscopy and SAXS. Finally, we determined that this lectin binds to the bacterial cell surface and elicits a proinflammatory response in cultured respiratory epithelial cell cultures. Together, we conclude that BC2L-C is a novel superlectin with multiple specificities and biological functions.

## Results

### BC2L-C consists of two distinct lectin domains

**Figure 1:**
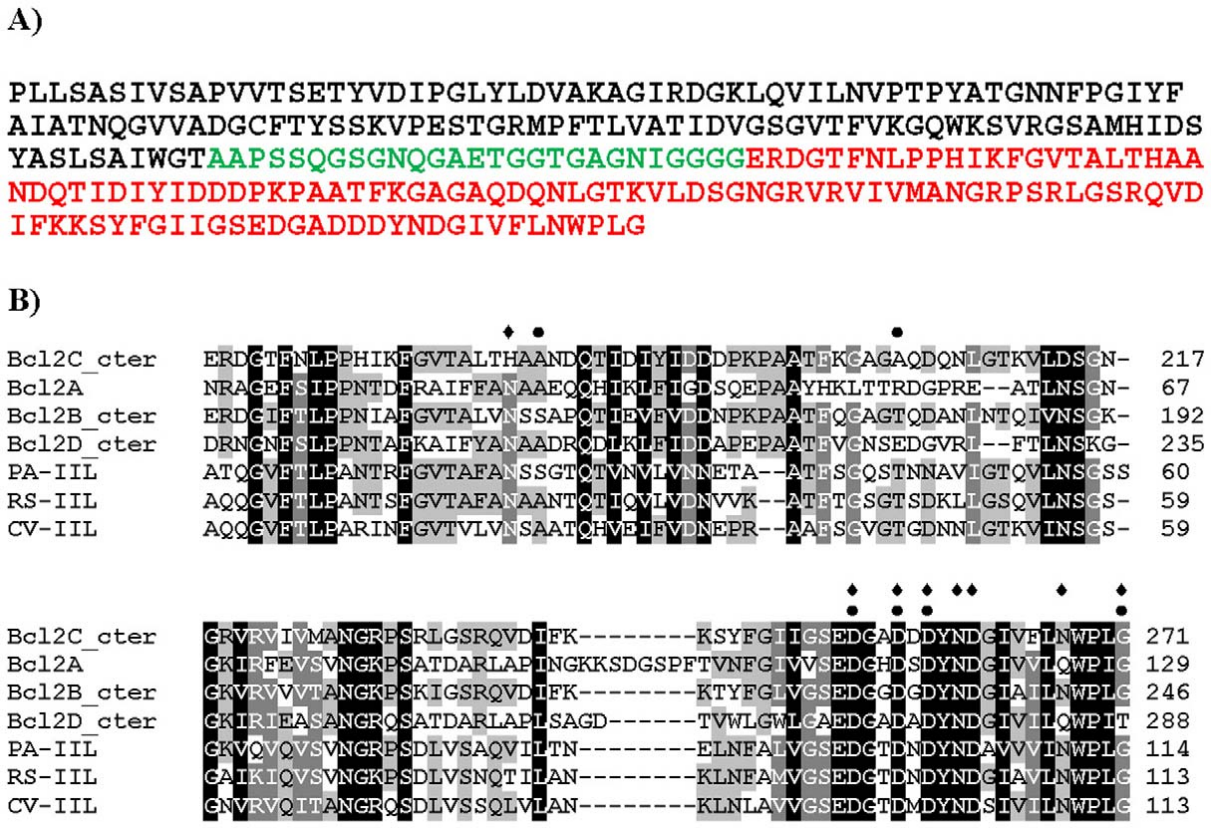
Sequence information. A) Peptide sequence of Bc2L-C with N-terminal domain in black, linker in green and C-terminal domain in red. B) Sequence alignment of the C-terminal domain of BC2L-C with related lectins from *Burkholderia cenocepacia, Pseudomonas aeruginosa, Ralstonia solanacearum* and *Chromobacterium violaceum*. Grey shading indicates the conservation of amino acids (black for fully conserved). Amino acids involved in calcium binding are indicated by diamonds. Amino acids predicted to form hydrogen bonds with the carbohydrate ligand are indicated by dots.

BC2L-C has an N-terminal region of 155 amino acids (the TNF-α-like lectin), a 28-aa linker region, and a 115-aa C-terminal region (Fig. 1A) [12]. The C-terminal domain has sequence similarity with two-calcium bacterial lectins such as LecB/PA-IIL from *P. aeruginosa* (43% identity) and related ones (Fig. 1B). The Ala-Ala-Asn sequence in the “specificity loop” [13] suggests that this domain is specific for mannose.

**Figure 2:**
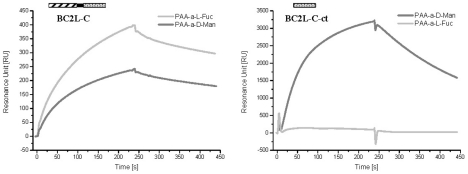
SPR sensorgrams of BC2L-C and C-ter domain on different monosaccharide-activated surfaces. The whole lectin binds efficiently to CM5 chips covered with PAA-fucose and PAA-mannose (left panel), while the isolated C-terminal domain has a strong specificity for fucose (right panel). In both cases, control curves obtained with galactose-modified channels have been subtracted.

BC2L-C interacts strongly with surfaces modified by mannose and fucose residues but not by galactose, as shown by Surface Plasmon Resonance (SPR) experiments, while the C-terminal domain, BC2L-C-ct binds only to mannose-coated chips (Fig. 2). Since the isolated BC2L-C-nt domain binds strongly to fucose but not to mannose and galactose [12], BC2L-C is therefore a novel type of lectin consisting of independent fucose and mannose-binding domains.

**Figure 3:**
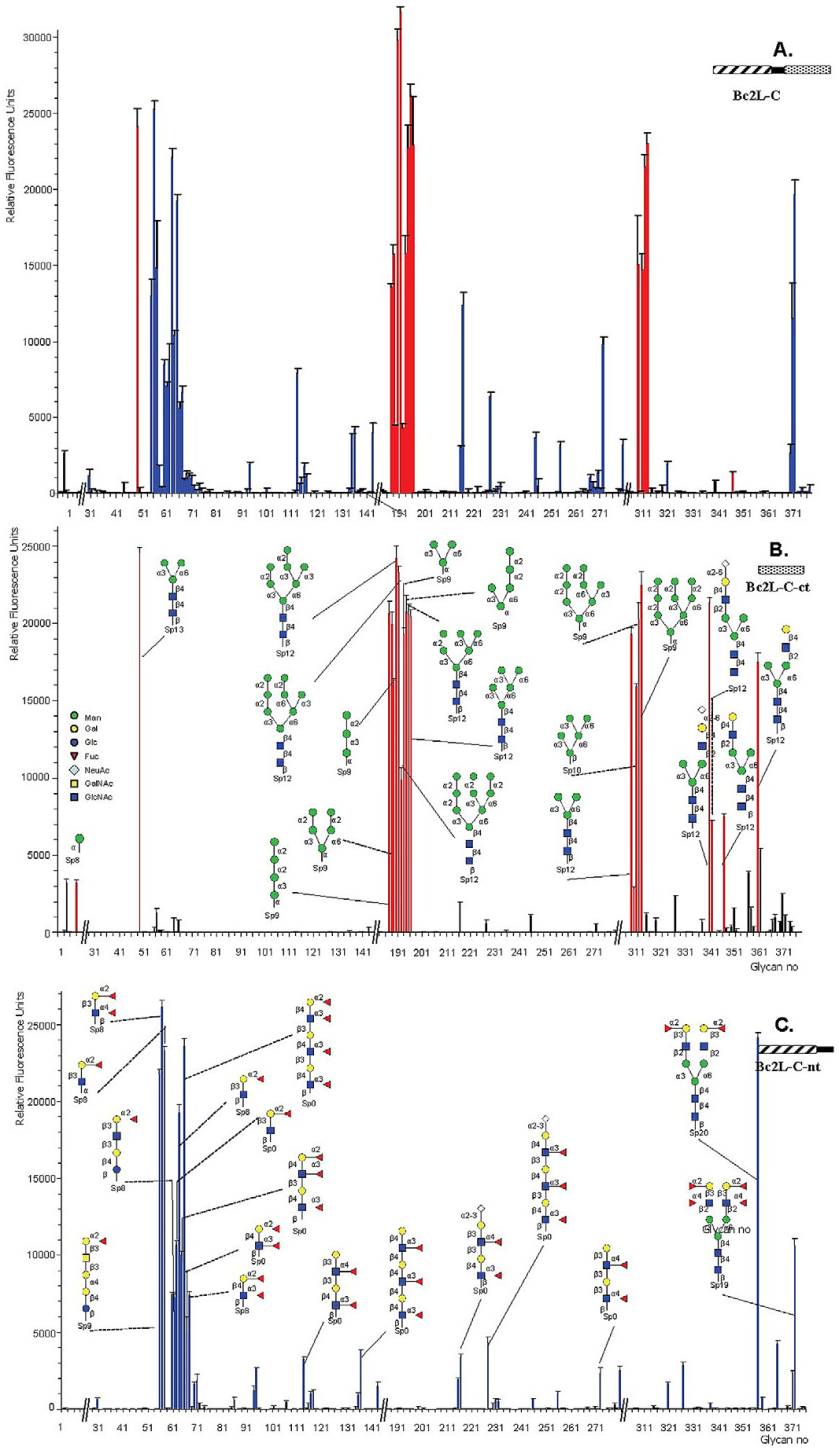
Glycan array data obtained with the whole BCL2-C lectin and the two domains expressed separately. Purified BC2L-C lectin samples were labeled with Alexa Fluor and tested on Glycan Array of the Consortium for Functional Glycomics. The BC2L-C-ct and BCLC-nt domains bind specifically to mannosylated and fucosylated oligosaccharides, respectively while the whole lectin binds to both.

The fine specificity of BC2L-C was determined using the Glycan Array facility of Consortium for Functional Glycomics with 377 carbohydrates available. BC2L-C bound to oligosaccharides containing terminal mannose or fucose residues (Fig. 3A). In contrast, the glycans bound by BC2L-C-ct included oligomannose-type N-glycans and their terminal fragments (Fig. 3B). The monosaccharide α-d-mannose (Man) was the shortest fragment recognized, albeit not efficiently (#8 in Fig. 3B). The recognised disaccharides were Manα1–2Man, Manα1–3Man and Manα1–6Man indicating that the specificity for the linkage is not strict. Hybrid structures with galactose or sialic acid on one antenna and α-mannose on the other one were also bound. The BC2L-C-nt domain is a fucosylated oligosaccharide binding lectin [12]. Therefore, bound fucosylated epitopes encompassed all fucosylated human histo-blood group epitopes such as blood group O(H) and Lewis oligosaccharides, with some preference for the Fucα1-2Gal epitope (Fig. 3C). The specificity charts for the separated domains do not overlap but their superimposition clearly explains that the specificity of the whole protein is determined by the contributions of the specificities of each domain.

### The C-terminal domain of BC2L-C is a calcium-dependent mannose/heptose binding lectin

The interaction of BC2L-C-ct with different carbohydrates was characterised by isothermal titration microcalorimetry. All thermograms display exothermic peaks with saturation of binding sites at the end of titration (Fig. S1). Affinity values and thermodynamics parameters are reported in Table 1. BC2L-C-ct bound to Man and α-methyl-mannoside (αMeMan) with a strong affinity in the micromolar range but with a stoichiometry close to 0.5, indicating that only one binding site per dimer is accessible. Using the whole protein, the same stoichiometry and affinity was measured for mannoside, but not for Lewis Y, a fucosylated oligosaccharide that binds to the other domain with a stochiometry of one (n = 1). The branched trimannoside (Manα1-3(Manα1-6)Man) exhibited an even lower stoichiometry (n = 0.22) demonstrating that the two terminal mannose residues bind to two BC2L-C-ct dimers, as observed previously for BC2L-A/trimannose interaction [11].

**Table 1:** Microcalorimetry titration data for the binding of monosaccharides and oligosaccharides to BC2L-C and BC2L-C-ct.

Ligand	n	*K_d_*(µM)	-ΔH (kJ/mol)	-TΔS (kJ/mol)
**BC2LC-C-ct**				
D-Man	0.50	37.4	57.0	31.8
αMeMan	0.52	27.6	55.0	29.0
Trimannose a	0.22	28.8	123.2	97.3
αMeHept	0.56	236	42.1	20.5
Diheptose b	0.48	88.1	39. 2	16.0
**BC2L-C**				
D-Man	0.57	21.8	6.1	34.5
αMeMan	0.49	18.3	73.5	46.4
Lewis Y	1.01	47.5	40.9	16.2

All measured values are averaged over at least two experiments. Standard deviations are <0.03 for n values and <5% for the other ones.

a trimannose: Manα1-3(Manα1-6)Man.

b diheptose: L,D-Hepα1-3L,D-Hep.

Binding was also tested towards l-*glycero*-d-*manno*-heptopyranose (Hept) since this residue is similar to mannose differing only in an additional hydroxymethyl group at C-6 and several Hept residues are in the *B. cenocepacia* lipopolysaccharide (LPS) [14], [15]. The methylated monosaccharide (αMeHept) bound with an affinity of 150 µM and the α1-3 linked disaccharide bound with an affinity of 88 µM, indicating that heptose-containing LPS could be a candidate substrate for BC2L-C-ct binding.

**Figure 4:**
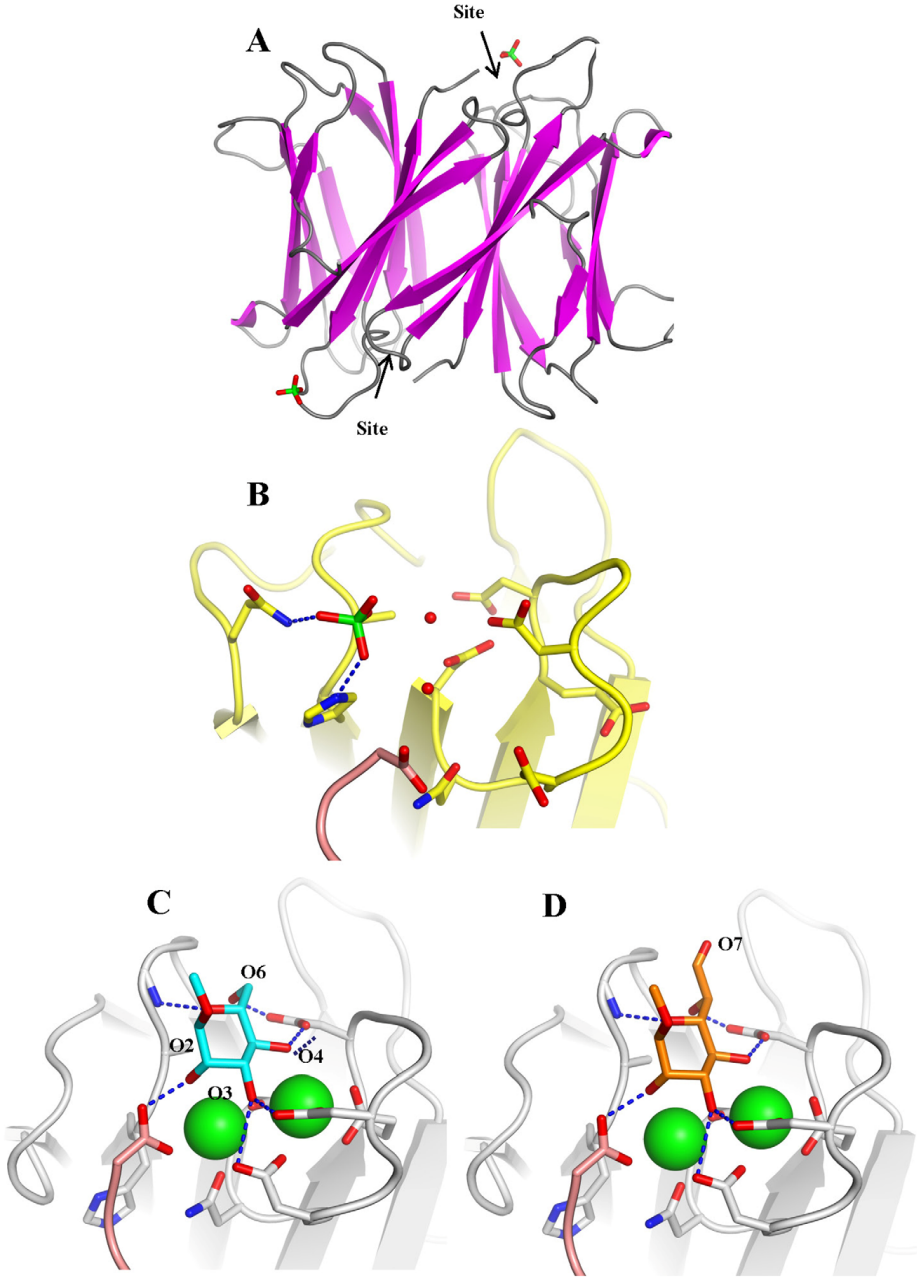
Crystal structure of apoBC2L-C C-terminal domain. A. Dimeric organisation of apo-BC2L-C-Ct, the protein is represented as ribbon. The sulphate ion close to one of the calcium and monosaccharide binding site is represented as sticks. B. Open conformation of the binding site in the absence of calcium and ligand. The sulphate ion interacts with Gln204, His177 and water molecules. C. Molecular modelling of BC2L-C-Ct in complex with calcium and α-methyl-mannoside with rearrangement of binding amino acids. D. Model of the complex with α-methyl-heptoside.

The crystal structure of BC2L-C-ct was solved at 1.9 Å resolution (Table 2), demonstrating a nine-stranded antiparallel β-sandwich fold that is similar to the two-calcium lectins characterised in *P. aeruginosa* [16], *Chromobacterium violaceum* [17], and *Ralstonia solanacearum* [18] (Fig. 4A). The dimeric association displays close similarity to the lectin BC2L-A, described previously [7], [11]. In contrast to other crystal structures in this family, no electron density for calcium ions and monosaccharide (d-mannose) was found in the final model, presumably due to the presence of citric acid in the crystallization buffer. Without the stabilizing effect of the two calcium ions in the binding site, the acidic amino acids that mediate their binding appear to point in all directions (Fig. 4B). A sulphate ion was observed close to one of the two binding sites, establishing hydrogen bonds with Gln241, His177 and two bridging water molecules.

**Table 2:** Data collection and refinement statistics of BC2L-C-ct crystal structure.

	native
***Data collection statistics***	
Beamline	ID14-1
Unit cell (Å)	*a* = *b* = 100.81*c* = 47.313
Spacegroup	P6_5_
Wavelength (Å)	0.934
Resolution limit (Å)	50.38–1.90 (2.00–1.90)*
Total observations	136740
Unique reflections	21277 (2700)
Completeness	97.5 (85.9)
Multiplicity	6.4 (2.4)
<I>/<σI>	11.3 (2.8)
R_merge_ (%)^b^	5.3 (21.1)
Wilson B-factor (Å^2^)	17.69
***Refinement statistics***	
R_cryst_	15.8%
R_free_	19.9%
RMSD bonds	0.015
RMSD angles	1.488
Ramachandran's outliers	2
Protein atoms	1795
Water atoms	286
Other atoms	10
*Overall B-factors*	
Main chains	16.6
Side chains	19.3
Water atoms	33.6
PDB code	2XR4

* Values in parenthesis refer to the highest resolution shell.

b Rmerge  =  ∑|*I*−<*I*>|/|∑<*I*>|, R*_cryst_*  =  (∑||*F_obs_* – *F_calc_*||)/(∑||*F_obs_*||).

Modelling the complex with αMeMan was possible since BC2L-C-ct has strong sequence similarity to the other lectins of the family (Fig. 1B), in particular with the *R. solanacearum* RS-IIL for which a crystal structure with calcium and mannose is available [18]. The modelled binding site was built by reorienting the amino acids side chains and slightly modifying the conformations of the loops (Fig. 4C). The main difference with the mannose-binding site of RS-IIL is the presence of His177 (Asn in all other lectins). In the absence of calcium, this histidine interacts with one sulphate ion but also modifies the conformation of the C-terminus of the other chain. Since the C-terminal carboxyl group has an essential role in binding mannose and calcium in all the other similar lectins, the putative destabilizing role of His177 could account for the observed non-even stoichiometry of the dimer. Modeling the interaction between BC2L-C-ct and αMeHept was achieved by extending the hydroxymethyl group at C5 of mannose in a glycolyl one. The binding site can accommodate this bulky group with no steric hindrance and the mode of binding of αMeHept displays the same hydrogen bond network that is observed for mannose (Fig. 4D).

### BC2L-C is a hexameric superlectin with dimeric and trimeric subdomains and flexible linkers

**Figure 5:**
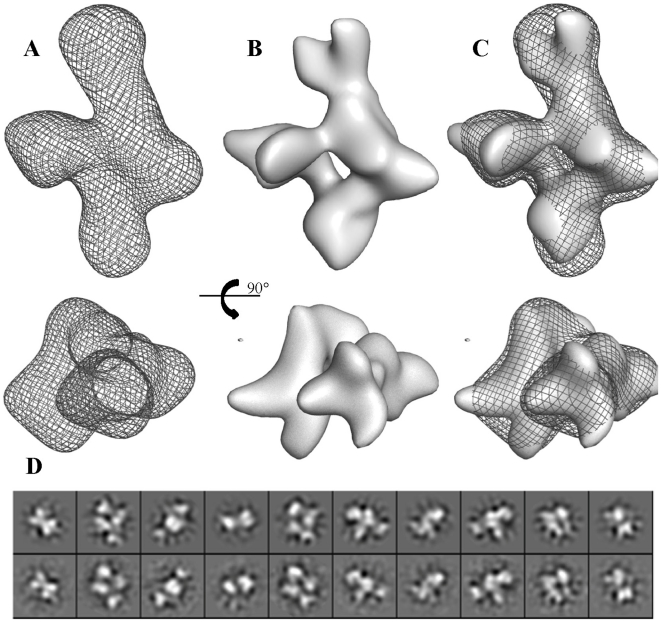
Three-dimensional reconstruction of BC2L-C. A. Refined ab-initio SAXS envelop. **B**. Isosurface representation of the EM reconstruction. C. Superposition of EM reconstruction with the SAXS envelope. D. Ten projections of the EM reconstruction (upper row) are shown for a visual comparison with the class averages (lower row).

The oligomeric state of BC2L-C and the C- and N-terminal domains were analysed by size exclusion chromatography combined with multi-angle laser light scattering (SEC-MALLS) and refractometry (RI) (Fig. S2). BC2L-C-ct is dimeric in solution with a molecular mass of 22±1 kDa whereas BC2L-C-nt is trimeric with a molecular mass of 41±1 kDa (the expected monomeric masses are 12.4 kDa and 19.3 kDa, respectively). The SEC/MALLS profile of the whole BC2L-C analysis indicates a hexamer in solution with a molecular mass corresponding to 145±4 kDa (the expected mass of the monomeric form is 28.2 kDa). These results are consistent with the crystallographic data of each domain; the dimeric C-terminal domain established here and the previously determined trimeric N-terminal domain [12]. The overall shape of the hexamer in solution was determined by small angle X-ray scattering (SAXS) and validated by negative stain electron microscopy (EM). The Guinier analysis (Fig. S3 and Table S1) suggests an *Rg* of ∼5 nm with the absence of severe aggregation effects that allowed for the *ab initio* shape reconstruction to be performed using the idealized SAXS curve. The refined *ab initio* envelope is elongated (max length ∼160 Å) with a pseudo 3-fold axis in the long direction and three bulges protruding from the middle of this long axis. (Fig. 5A). Negative stain electron microscopy analysis validated the SAXS results. Indeed, the three-dimensional reconstruction of BC2L-C at 20 Å resolution (Fig. 5B and 5D) shows the same global shape as the SAXS envelope (Fig. 5C). These complementary results confirmed the size and overall shape of the molecule allowing manual fitting of the domains using the combined EM and SAXS reconstructions as the template.

**Figure 6:**
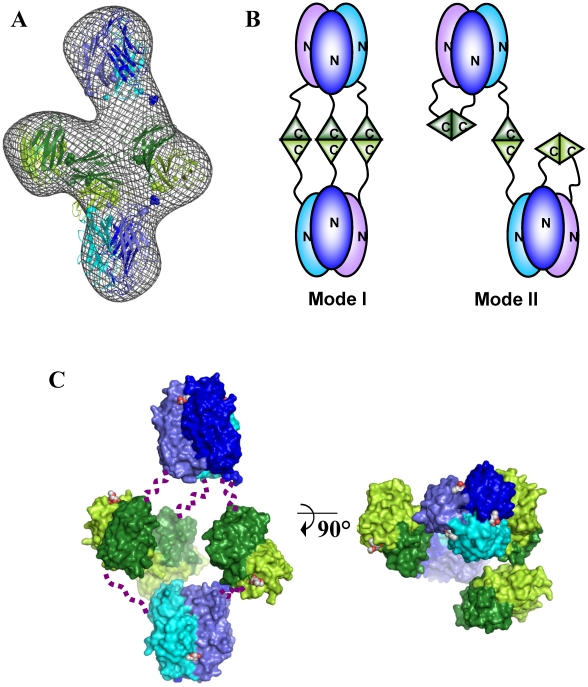
Models of the BC2L-C hexamer with N-terminal domains in blue and C-terminal domains in green. A. Best manual fit of the different domains of BC2L-C hexamer in the *ab-initio* SAXS envelop. B. Two possible arrangements of trimers that could correspond to the manual fit. C. Orthogonal orientations of the mode I. Linkers have been schematized by dotted lines. Calcium and carbohydrate ligands (fucose for the N-terminus and mannose for C-terminus) are represented by spheres.

Positioning two trimers of BC2L-C-nt on the large axial bulges and three dimers of BC2L-C-ct on the equatorial ring-like envelop fitted well within the envelope (Fig. 6). However, attempts to mathematically optimize this model were partially successful probably because of the absence of the linker moieties in the model. By adding random chains for the missing linkers (6 chains each 28 residues in length) an acceptable value of chi = 3.5 could be attained (Fig. S4). Distances between domain extremities were checked in the final model, the maximum one being 50 Å, a value that allow for the 28 amino acid linkers to fit. The positions of the linkers are not presented as definitive as the entire complex appears to be flexible, especially in the central part of the molecule. Our results suggest that the low-resolution shape with the two trimers separated along the long axis and three dimers in the middle is the conformation adopted in solution under physiological conditions. A general 3-fold axis is visible, passing by through the trimers and by the center of the donut, but the symmetry is broken by the twisted orientations of dimers. Since the linkers could not be located using available methods, two possible architectures (mode I and mode II) can be proposed for the BC2L-C hexamer (Fig. 6B). However, our shape reconstruction indicates that mode I is more probable to occur in solution as the mode II would generate more extended structures of high conformational variability.

### BC2L-C is immunogenic and located at the bacterial surface

**Figure 7:**
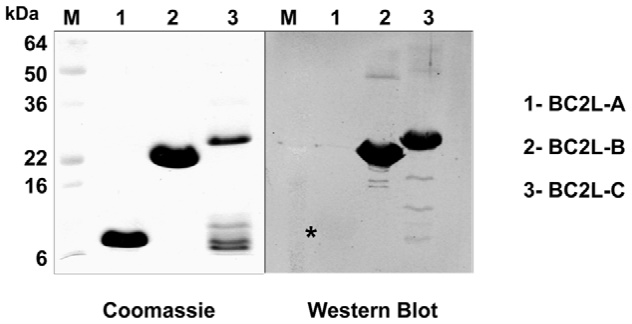
Immunodetection of soluble lectins. 10 µg of purified lectins were separated on a 16% SDS-PAGE, and the gel stained with Coomassie Blue or transferred to nitrocellulose membranes that were reacted with anti-cepacia polyclonal rabbit antibodies. Reacting bands were detected by fluorescence with an Odyssey infrared imaging system (Li-cor Biosciences) using IRDye800CW affinity purified anti-rabbit IgG antibodies (Rockland, Pennsylvania). M, prestained molecular weight standards. The star indicates the presence of a weak bank for Bcl2L-A.

The expression of BC2L-C in *B. cenocepacia* J2315 was previously demonstrated by classical proteomics [12]. Western blots were performed with the purified recombinant lectins BC2L-A, -B and -C using rabbit antisera prepared against formalin-fixed intact *B. cenocepacia* K56-2 cells (clonally related to J2315). The purified recombinant lectins BC2L-B and -C are strongly detected by anti-K56-2 antibodies, while BC2L-A is barely detectable (Fig. 7). This result agrees with the observed expression levels of BC2L-A, -B, and -C lectins in *B. cenocepacia* K56-2. Since intact bacterial cells were used in the immunisation procedure, we conclude that the lectins are present on the cell surface.

**Figure 8:**
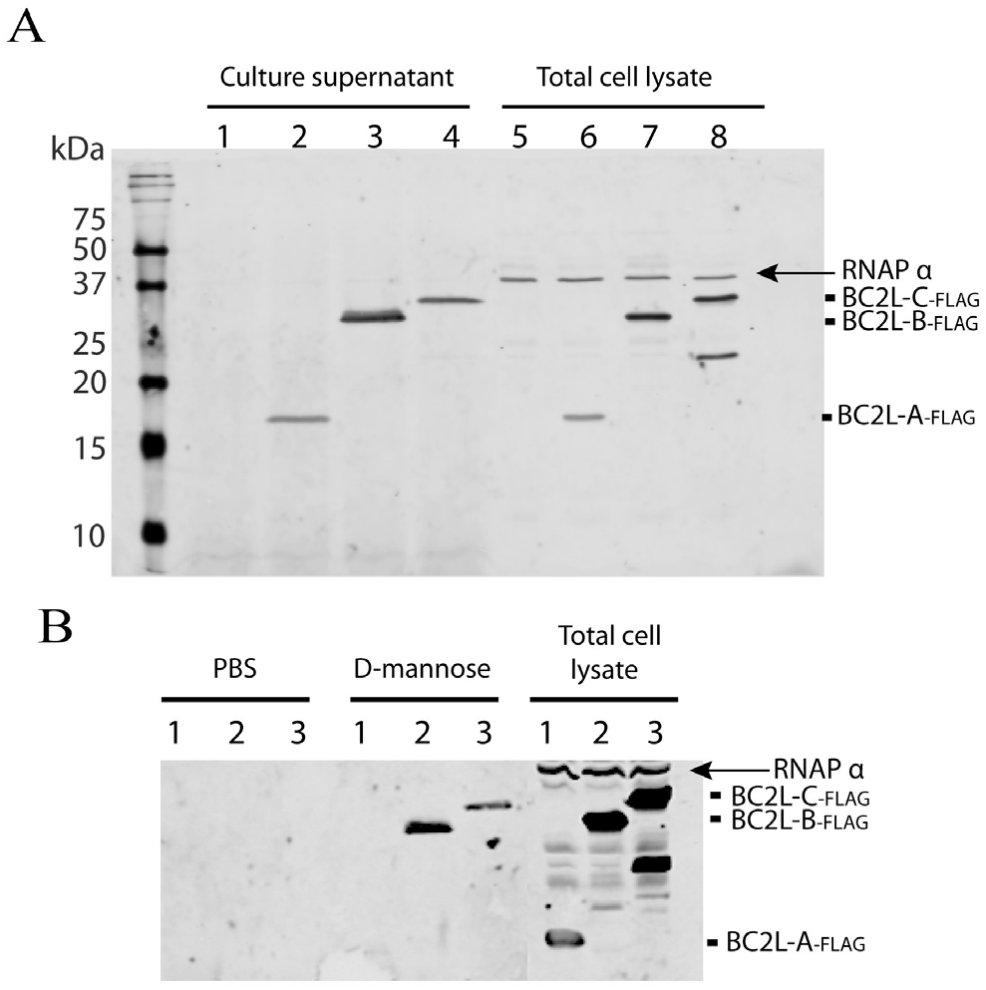
Localisation of *B. cenocepacia* soluble lectins in bacterial cells. A. BC2L-A, -B and –C are secreted into the growth medium. Analysis of concentrated culture supernatants and total cell lysates recovered from *B. cenocepacia* K56-2 containing the control plasmid pEL-1 (lanes 1 and 5), K56-2 pBC2L-A_-FLAG_ (lanes 2 and 6), K56-2 pBC2L-B_-FLAG_ (lanes 3 and 7) and K56-2 pBC2L-C_-FLAG_ (lanes 4 and 8). B. BC2L-B and –C are released from the bacterial surface upon mannose treatment. Analysis of concentrated supernatants from cells treated with PBS or 50 mM d-mannose and total cell lysates recovered from *B. cenocepacia* K56-2 pBC2L-A_-FLAG_ (lanes 1), K56-2 pBC2L-B_-FLAG_ (lanes 2) and K56-2 pBC2L-C_-FLAG_ (lanes 3). Western blots were performed using anti-FLAG and anti-RNAP alpha subunit antibodies (cell lysis control). A degradation product from BC2L-C can be detected in the total cell lysates. The arrow highlights the position of the RNAP alpha subunit in total cell extracts. Samples were boiled 10 min prior to loading on 18% SDS-PAGE gels.

BC2L-A, -B and -C lectins were tagged with a FLAG epitope at their N-terminus and expressed as recombinant lectins in *B. cenocepacia* K56-2. Culture supernatant analysis by Western blot using anti-FLAG and anti-RNA polymerase alpha subunit antibodies (used as a cell lysis control) revealed that the three lectins are secreted or released into the extracellular medium without detectable cell lysis (Fig. 8A).

To determine whether BC2L-A, -B and C lectins associate to the bacterial cell surface, bacterial cells expressing the FLAG-tagged lectins were incubated with buffer or with 50 mM d-mannose for 5 min. Western blot using anti-FLAG and anti-RNAPα revealed that BC2L-B and -C lectins are released into the supernatant upon incubation with mannose but not with buffer only. That BC2L-B and -C lectins are specifically released upon mannose treatment without any detectable cell lysis suggests that BC2L-B and -C lectins are located on the surface of *B. cenocepacia* (Fig. 8B).

### The BC2L-C TNF-α-like N-terminal domain elicits IL-8 secretion by epithelial cells

**Figure 9:**
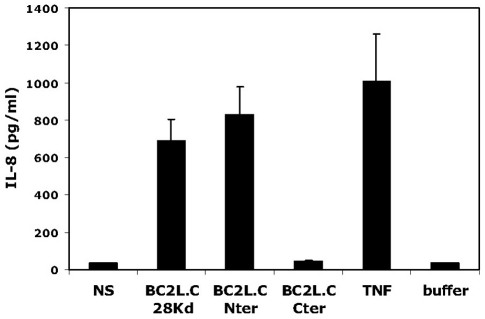
Activation of respiratory epithelial cells by BC2L-C and its separate domains. Sub-confluent BEAS-2B cells cultured in 24-well plates were incubated in 300 µL medium with either BC2L-C, BC2L-C-nt or BC2L-C-ct at 0.3 µM. As negative and positive controls, cells were either not stimulated (NS), incubated with the suspension buffer of BC2L-C (buffer) or challenged with 10 ng/mL of TNF-α (TNF). After 15 h, supernatants were collected and IL-8 concentrations were measured by ELISA. Each histogram is the mean ± sem of 3 experiments performed in triplicate.

Since the crystal structure of BC2L-C-nt demonstrated a TNF-α-like fold [12], the immunostimulatory activities of BC2L-C and its domains were tested on epithelial cells. A markedly increase in IL-8 production was observed in cells exposed to BC2L-C and this activity was attributed to the N-terminal domain (Fig. 9). Attempts to inhibit the IL-8 production by carbohydrate ligands were unsuccessful (data not shown), indicating that the carbohydrate-binding and pro-inflammatory eliciting activities reside in different parts of the molecule. Attempts to inhibit IL-8 production using siRNA directed against the TNF-α receptor (TNFR1) also resulted in negative results (Fig. S5). The immunostimulatory activity of BC2L-C is therefore mediated by its N-terminal domain in a carbohydrate-independent manner, and does not appear to be mediated by selective binding to TNFR1.

## Discussion

To our knowledge, BC2L-C is the first protein identified harbouring two different lectin domains with distinct specificity, for which we refer to it to as a “superlectin”. Multispecificity was only found in lectins with duplicated domains resulting from divergent evolution, such as some human galectins and plant lectins [19], [20]. The association of two functionally and structurally distinct domains in BC2L-C is therefore the paradigm for a new class of lectins.

The cellular localisation of the soluble lectins produced by opportunistic bacteria remains an open question. While the lectins have a role in host recognition, they are present in large quantity in the cytoplasm and do not contain any canonical secretion signals. Previous work demonstrated the location of LecB on the outer membrane of *P. aeruginosa* [21] and recent work suggested that transient glycosylation of the lectin is required for transportation [22]. Our data indicate that *B. cenocepacia* lectins are also located at the bacterial surface. Control data monitoring the RNA polymerase alpha subunit (cytoplasmic protein used as cell lysis control) demonstrate that the lectin does not exit by simple cell lysis. Therefore, the lack of typical secretion sequences in these lectins suggests they are secreted by one or more specialized secretion systems that are yet to be identified. We also demonstrate that surface localisation depends on the mannose-binding site in the C-terminal domain, since treatment of bacterial cells with d-mannose results in the release of the lectins. Since this binding site has strong affinity for l-d-heptose, an abundant component of the *B. cenocepacia* LPS [14], it is possible that LPS may provide an attachment site on the bacterial surface. However, attempts to demonstrate lectin binding to LPS were unsuccessful (data not shown), suggesting that the lectin may bind to a different bacterial surface molecule.

**Figure 10:**
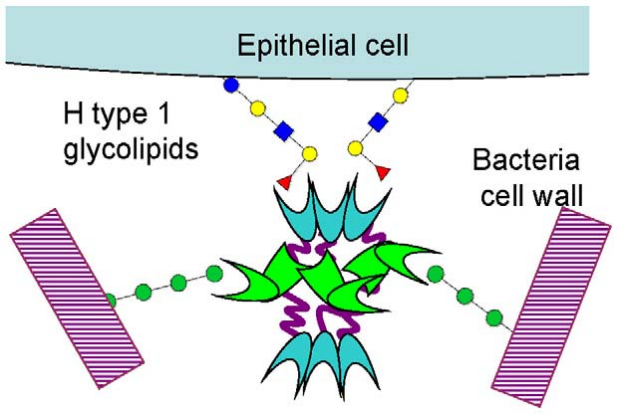
Schematic representation of BC2L-C hexamer cross-linking host epithelial cells and bacteria surface.

The unique hexameric architecture of BC2L-C is well suited for cross-linking between bacteria and epithelial cells (Fig. 10). The advantage of such flexible structure is that all carbohydrate binding sites can be exposed at the surface and free to interact. Also, a flexible linker could adapt its conformation under shear force and provide tight binding as observed in some pili adhesins [23]. The three mannose/heptose binding sites, responsible for bacterial surface binding, are located in the external part of the middle ring, while the fucose binding sites, that binds to H-type 1 and other fucosylated epitopes on glycolipids, are present at each extremity. These TNF-α-like N-terminal domains have a strong pro-inflammatory effect, as determined by IL-8 release by epithelial cells. Lung infection by *B. cenocepacia* in CF patients is characterized by strong inflammation [24]. In addition to the classical activation of Toll-like receptors by LPS and flagella [25], it has been recently demonstrated that *B. cenocepacia* activates the TNFR1 signalling in cystic fibrosis airway epithelial cells [26].

In conclusion, our study opens many questions about the biological function of super lectins in opportunistic bacteria. Future work will unravel the binding epitope on the bacterial cell surface and provide more details on the physiological role of the super lectin in the infection processes.

## Materials and Methods

### Carbohydrate material

Monosaccharides (Sigma), trimannoside (Dextra) and Lewis Y (Elicityl) were obtained from commercial sources. Methyl l-*glycero*-α-d-*manno*-heptopyranoside and allyl l-*glycero*-α-d-*manno*-heptopyranosyl-(1→3)-l-*glycero*-α-d-*manno*-heptopyranoside were synthesized according to published procedures [27], [28] that are briefly described in Text S1.

### Gene cloning and protein expression and purification

The gene encoding full-length BC2L-C was synthesized by GenScript Corp with optimization for expression in *E. coli* and contained flanking NdeI and HindIII sites. This synthetic gene was cloned into pRSET vector (Invitrogen), resulting in pRSET*_bc2l-c*, which was used as a template to clone pRSET_*bc2l-c-ct* encoding BC2L-C-ct (Table S2). *E. coli* BL21 (DE3) cells containing plasmid pRSET*_bc2l-c* and/or pRSET*_bc2l-c-ct* were cultured in LB broth low salt medium (Duchefa Biochemie) containing 100 µM ampicillin at 37°C until the OD_600_ reached ∼0.5. After the addition of 0.5 mM IPTG (Duchefa Biochemie), cells were cultured for an additional 3 hours at 30°C, harvested by centrifugation and resuspended in 20 mM Tris buffer containing 100 mM NaCl and 100 µM CaCl_2_, pH 7.5. Cells were disintegrated by ultrasonic vibration and the soluble fraction was collected by centrifugation at 21000 g at 4°C for 30 min. Harvested cells were stored in plastic falcons at −20°C. Recombinant BC2L-C and/or BC2L-C-ct were purified by affinity chromatography on the mannose-agarose (Sigma-Aldrich) column using an FPLC system (ÄCTA, GE Healthcare). After washing, specific elution was carried out with 20 mM Tris pH 7.5, 100 mM NaCl and 10 mM EDTA. The protein was dialysed in 10 mM Tris pH 7.5, 20 mM NaCl and 1 mM CaCl_2_. Protein purity was assessed by SDS-PAGE (12% gel) and stained with Coomassie Brilliant Blue R-250 (Sigma Aldrich). Preparation of BC2L-C-nt was performed as described previously [12].

### Surface plasmon resonance

SPR experiments were performed on a BIAcore 3000 instrument (GE Healthcare) at 25°C using a running buffer HEPES - Buffered Saline (HBST) (10 mM HEPES and 150 mM NaCl, pH 7.5 containing 0.005% (v/v) Tween 20) and a flow rate of 5 µL per minute. Two different chips presenting monosaccharides have been used. Biot-PAA (biotinylated polyacrylamide) probes bearing sugar moieties (Lectinity Corp, Russia) were trapped on a CM5 (BIAcore Life Science) sensor chip that was coated with streptavidin using the standard procedure (Amine coupling, BIAcore Sensor Surface Handbook). Each Biot-PAA-monosaccharide (50 µL at concentration 200 µg/mL) was injected to the selected channel. Direct binding curves of the lectin to immobilised sugars were measured over the concentration range 0.35–0.45 mg/mL. Samples were injected (20 µL, KINJECT) onto the CM5 chip at a flow rate 5 µL/min. The chip was regenerated using 100 mM EDTA and 50 mM NaOH. Binding of the proteins to the immobilised sugars was determined by resonance units (RU) over time and data were evaluated using the BIAevaluation Software (version 4.1).

### Glycan array

Purified BC2L-C lectin samples were labeled with Alexa Fluor 488-TFP (Invitrogen, CA) according to manufacturer's instructions and re-purified on a D-Salt polyacrylamide desalting column (Pierce, Rockford IL). Alexa-labeled proteins were used for glycan-array screening with standard procedure of the Core H of the Consortium for Functional Glycomics (Emory University, Atlanta, GA, http://www.functionalglycomics.org). The screening of the printed glycan microarray chip (version 3.2, with 377 glycans from a library of natural and synthetic glycans) was performed with a concentration of BC2L-C of 200 µg/mL dissolved in 20 mM HEPES, 140 mM NaCl, 5 mM CaCl_2_, pH 7.5 for all samples.

### Microcalorimetry

Experiments were performed at 25±0.1°C using VP-ITC and ITC_200_ calorimeters (Microcal, GE Healthcare). Saccharides and proteins were dissolved in the same buffer (20 mM Tris pH 7.5, 20 mM NaCl and 0.03 mM CaCl_2_). Protein concentration for measurements varied from 125 to 400 µM. Aliquots of 2 or 10 µL of sugar solution at various concentrations from 1.56 to 50.0 mM, were added automatically to the protein solution present in the calorimeter cell. Stirring was adjusted to at 300 and 1000 rpm. Titration of BC2L-C and BC2L-C-ct was performed with αMeMan, d-mannose and α-methyl-l-fucoside, trimannose and diheptose. Control experiments performed by injections of buffer in the protein solution yielded insignificant signals. Integrated heat effects were analysed by non-linear regression using a single-site binding model (Microcal Origin 7). The experimental data fitted to a theoretical titration curve brought up the association constant *K_a_* and the enthalpy of binding *ΔH.* The other thermodynamic parameters such as free energy *ΔG* and entropy *ΔS* were calculated from the equation: *ΔG  =  ΔH - TΔS  =  -RTlnK_a_*, where *T* is the absolute temperature and *R* is molar gas constant (8.314 J.mol^−1^.K^−1^). All experiments were performed with *c* values between 10< *c* <100 [29]. At least two or three independent titrations were performed for each tested ligand.

### Crystallography and molecular modelling

Lyophilized BC2L-C-ct was solubilised (14.5 mg/mL) in 5 mM Tris buffer (pH 7.5) containing 4.0 mM αMeMan and 0.1 mM CaCl_2_. Initial crystallization conditions were determined using commercial crystallization screens (Hampton Research and Molecular Dimension Limited) using a Mosquito robot (TTP LabTech Ltd). Protein crystals in the form of thin baguettes with hexagonal profile appeared after several weeks at 17°C in the presence of 100 mM Sodium Citrate pH 5.5 and 2.5 M Ammonium Sulphate. These initial conditions were optimized and scaled up to 4 µL hanging drops resulting in bigger crystals with the same shape. Crystals were cryocooled at 100 K in liquid nitrogen after soaking them for as short a time as possible in 30% (v/v) glycerol mixed with precipitant solution. Diffraction data for BC2L-C-ct were collected on the beamline ID14–1 at ESRF (Grenoble) using an ADSC Q210 CCD detector (Quantum Corp.). Diffraction images were integrated using MOSFLM [30], scaled and converted into structure factors using the CCP4 program suite [31]. Protein crystallised in the hexagonal space group *P*6_5_ (*a*  =  *b*  = 100.814 Å, *c* = 47.313 Å, γ = 120.0°) with two monomers in the asymmetric unit. The 1.9 Å structure was solved by molecular replacement using the MOLREP program [32], [33]. A monomer of CVIIL lectin (PDB: 2BV4) from *Ch. violaceum* [17] was used as a search model. Crystallographic refinement was carried out with the program REFMAC5 [34] alternated to manual rebuilding using WinCoot [35]. The solvent model was built automatically with the program ARP/wARP [36] and revised manually with WinCoot. Stereochemical verification was performed with the PROCHECK program [37]. Details about data collection and refinement statistics are available in Table 2. The final model for the apo-form of BC2L-C-ct was deposited in the PDB database with accession code 2XR4. A model of the binding site in complex with αMeMan and calcium ions was produced combining the *-apo* structure from the present structure combined with that from the complex between *R. solanacearum* RS-IIL and αMeMan (pdb code 1UQX) [18]. Briefly, a monomer of RS-IIL complexed with αMeMan was fitted on one monomer of BC2L-C-ct and the side chains of amino acids in the binding site of BC2L-C-ct were adjusted to match those of RS-IIL. Coordinates for monosaccharide and calcium ions were merged with those of BC2L-C-ct. Hydrogen atoms and partial charges were added using Sybyl software (Tripos Inc, St Louis) using Amber parameters for the protein and PIM parameters for carbohydrates [38]. Energy minimisation was performed with geometry optimisation of all hydrogen atoms, monosaccharide and side chains in the binding site. Graphical representations are performed with Sybyl and Pymol (Pymol.org).

### Size exclusion chromatography - Multiple angle laser light scattering (SEC-MALLS)

BC2L-C whole protein and its separate domains were analysed on the Superdex 200 (GE Healthcare) column equilibrated with 20 mM Tris, 250 mM NaCl, 1 mM CaCl_2_, pH 7.5 using the FPLC system (ÄCTA, GE Healthcare). A 200 µL sample was loaded at a flow rate of 0.4 mL/min. Molecular weights were determined using gel filtration standard (Bio-Rad). Fractions corresponding to molecular mass of hexamer, dimer and trimer, respectively, were concentrated by centrifugation (Vivaspin, Sartorius Stedim Biotech) up to the concentration of 4.2 mg/mL for BC2LC, 7.5 mg/mL for BC2L-C-nt and 10.7 mg/mL for BC2L-C-ct, respectively, and used for SEC-MALLS analysis. 100 µL of each sample was loaded at a flow rate of 0.4 mL/min. On-line MALLS detection was performed with a DAWNEOS detector (Wyatt Technology Corp.) using a laser emitting at 690 nm and by refractive index measurement using an RI2000 detector (Schambeck SFD). Weight-averaged molar masses (Mw) were calculated using the ASTRA software (Wyatt Technology Corp.).

### Small angle X-ray scattering

The BC2L-C protein was purified by gel filtration (as previously described) immediately prior to the SAXS experiment. The central fraction of the peak containing the hexameric BC2L-C (1.27 mg/mL) was collected and used to prepare two additional dilutions (0.66 and 0.31 mg/mL) with sample concentrations verified using a spectrophotometer (NanoDrop Technologies). The rest of the fractions containing the hexameric BC2L-C were collected and concentrated to 4.2 mg/mL. SAXS data from the resulting samples (from 0.33 to 4.2 mg/mL) were collected at the ESRF BioSAXS station (ID14EH3, http://www.esrf.fr/UsersAndScience/Experiments/MX/About_our_beamlines/ID14-3) at fixed energy wavelength (13.32 keV, λ = 0.931 Å). Samples were exposed using 30 µl of protein solution loaded into a 2 mm quartz capillary mounted in vacuum using an automated robotic system (developed as part of a trilateral collaboration between ESRF and EMBL Hamburg and Grenoble Outstation) which enables the sample to pass through the beam during exposure to minimise the effect of radiation damage. 2D scattering images were collected on a Pilatus 1M detector (Dectris) 1.83 m from the sample. Standard data collection was used for all data (10 frames each 10 second in duration). Individual time frames are processed automatically and independently by the data collection software (BsxCUBE) developed at the ESRF, yielding individual radially averaged curves of normalised intensity versus scattering angle (s = 4πSinθ/λ in nm). Time frames are combined excluding any data points affected by aggregation induced by radiation damage to give the average scattering curve for each measurement. The scattering from the buffer alone was measured before and after each sample measurement and the average of the scattering before and after each sample was used for background subtraction, the different concentrations were then compared and merged to obtain the idealized scattering curve using the program PRIMUS (13) form the ATSAS package developed by EMBL Hamburg. Ab-initio models were produced with DAMMIF (14) and averaged with DAMAVER [39]. Rigid body modeling was undertaken using MASHA [40] with 6 additional random chains of 28 residues created by ranch13 (also part of the ATSAS package from EMBL-Hamburg) to represent the linkers. The plot of the fits was produced with the beta version of SASPLOT from the upcoming cross-platform release of the ATSAS package developed at EMBL-Hamburg.

### Electron microscopy

For preparation of negatively stained BC2L-C, the purified sample was diluted to 0.05 mg/mL, applied to the clear side of carbon on a carbon-mica interface and stained with 2% (w/v) sodium silicotungstate at pH 7. Images were recorded under low-dose conditions with a JEOL 1200 EX II microscope at 100 kV and at nominal 40000× magnification. Selected negatives were digitized on a Zeiss scanner (Photoscan TD) at a step size of 14 micrometer giving a pixel size of 3.5 Å at the specimen level. A generous semi-automatic particle selection with the EMAN boxer routine [41] lead to an extraction of a total of 18426 subframes of 56×56 pixels containing individual BC2L-C complex particle frames which were CTF-corrected with CTFFIND3 [42] and bsoft [43], and low-path-filtered at 15 Å with Imagic-5. Subsequent data processing was performed with the Imagic-5 software package [44]. The data set was translationally but not rotationally aligned relative to the rotationally averaged total sum of the individual images. This translationally centered data set was subjected to multivariate statistical analysis and classification. Characteristic class averages were then used as a set of references for multireference alignment of each subframe with Spider [45], [46] and the new translational parameters were used to update the boxer coordinates and extract better centered particles. This procedure was repeated several times until the classes became stable and the individual frames well centered. At this point, the class averages were compared to projections of the current SAXS model of BC2L-C filtered to 25 Å resolution. Given a notable similarity between them, the SAXS model was filtered to 80 Å resolution in order to resemble a nearly featureless blob of density but conserve the particle dimensions. This blob was used as an initial model for iterative projection matching with Spider [45], [46]. The resolution of the final 3D reconstruction of the negatively stained BC2L-C was estimated via Fourier shell correlation to be around 20 Å according to the conservative 0.5 criterium.

### Production of FLAG-tagged lectins in *B. cenocepacia*

The epitope FLAG-containing sequence was excised from plasmid pBADNTF [47] and subcloned into pDA12 [48] using EcoRI and HindIII restriction enzymes, giving rise to pEL-1. The lectin encoding genes (*BCAM0184* (*bc2l-b*), *BCAM0185* (*bc2l-c*) and *BCAM0186* (*bc2l-a*)) were PCR amplified by use of *B. cenocepacia* J2315 genomic DNA as template and sense and antisense primers with BamHI and HindIII restriction sites, respectively that were designed for each gene. Primer pairs were as follows: (5′TTTAGGATCCTGCTGATTCTCAAACGTCATCCA-3′) and (5′-TTTTAAGCTTAACGTG CGTCAGGTCAGC-3′) for *bc2l-a*; (5′-TTTTGGATCCTTCACAACCCTTTACCCACGA-3′) and (5′-TTTTAAGCTTGTGATGTAACGGCGAAGACC-3′) for *bc2l-b*; (5′-TTTTGGATCCTC CCCTCCTTTCGGCTTCGAT-3′) and (5′-TTTTAAGCTTGTACAGCAGTGGGACTGCAA-3′) for *bc2l-c*. Amplicons were digested with BamHI and HindIII and ligated into similarly digested pEL-1 giving rise to pBC2L-A_FLAG_, pBC2L-B_FLAG_ and pBC2L-C_FLAG_ plasmids, which encode BC2L-A, BC2L-B and BC2L-C, respectively N-terminally fused to the FLAG epitope. Plasmids were mobilized into *B. cenocepacia* K56-2 by triparental mating using *E. coli* DH5α carrying the helper plasmid pRK2013 [49] as previously described. Exconjugants were selected onto tetracycline 100 µg/ml and gentamicin 50 µg/mL containing plates.

### Preparation of culture supernatant proteins and western blot analysis

Culture supernatant proteins were precipitated with trichloroacetic acid as described previously [48] The protein concentration was determined by Bradford assay (Bio-Rad) and 4 µg of protein were loaded on a 18% SDS-PAGE gel. After electrophoresis, gels were transferred to nitrocellulose membranes for immunoblot analysis. The membranes were incubated with the 4RA2 monoclonal antibody (Neoclone) cross-reacting with the *B. cenocepacia* RNA polymerase subunit alpha (cytosolic protein, cell lysis control) and the FLAG M2 monoclonal antibody (Sigma). The Alexa Fluor 680 goat anti-mouse IgG (Molecular Probes) was used as a secondary antibody. Detection was performed using the Odyssey Infrared Imager (LI-COR Biosciences).

### Mannose-dependent lectin extraction

Overnight cultures were diluted to an OD600 nm of 0.03 in 50 mL LB and grown at 37°C for 8 h. Cells were then centrifuged at 5000 g for 10 min. The pellet was washed twice with 25 mL of phosphate buffered saline (PBS) and finally resuspended in 1.5 mL PBS. Five hundred µL aliquots were placed into two eppendorf tubes to which 500 µL of PBS or 500 µL of 100 mM d-mannose made in PBS (50 mM final concentration) was added. Samples were gently mixed by inversion and incubated for 5 min at room temperature. Samples were centrifuged at 6000 g for 5 min, supernatants were collected (800 µL) and filter-sterilized using 0.2 µM filters. Proteins were precipitated overnight at 4°C with trichloroacetic acid (10% final concentration). Samples were centrifuged at 16 000 g for 30 min at 4°C. Each pellet was then washed with 1 mL of ice-cold acetone, air-dried and resuspended in 15 µL of sodium phosphate buffer 0.1 M pH 7.2. The totality of the samples were loaded on a 18% SDS-PAGE gel.

### Evaluation of pro-inflammatory activity

BC2L-C and its separate domains were used for the stimulation of the human bronchial cell line BEAS-2B obtained from the American Type Cell Collection (Manassas, VA). Cells were maintained in serial passage in F-12K culture medium supplemented with 10% FCS, 1% penicillin and streptomycin, 1% glutamine and 10 mM HEPES in 75 cm2 culture flasks and seeded at 5×10^4^ on 24-well plates 3 days before stimulation. In all experiments, BEAS-2B cells were stimulated during 15 hours with the different agonists in a 300 µL medium. IL-8 concentrations in cell culture supernatants were determined using a Duo-Set ELISA kit. Duo-Set ELISA kit and the recombinant human TNF-α were obtained from R&D Systems (Minneapolis, MN).

## Supporting Information

Figure S1 **Microcalorimetry data.** (A) ITC plot (measured by VP-ITC, Microcal) obtained from the titration of Met-α-Man (3.036 mM) to BC2L-C-ct domain (386 µM). (B) ITC plot (measured by ITC200, Microcal) obtained from the titration of Trimannoside (2.937 mM) to BC2L-C-ct (510 µM). (C) ITC plot (measured by ITC200, Microcal) obtained from the titration of diheptose (2.1 mM) to BC2L-C-ct (350 µM). Protein and saccharide were prepared in 20 mM Tris pH 7.5, 100 mM NaCl and 5 mM CaCl_2_. Temperature 25°C was adjusted. The lower plots show the total head released as a function of total ligand concentration for the titration shown in panel up. The solid line represents the best least-square fit for the experimental data.
(DOCX)

Figure S2 **SEC-MALLSdata.** Size exclusion chromatogram combined with MALLS molecular mass evaluation of the whole protein BC2L-C (black curves), N-terminal domain (blue curves) and C-terminal domain (red curves). Short curves represent the molecular mass variation across the chromatographic peak.
(DOCX)

Figure S3 **Guinier analysis.** Calculated Guinier regions (straight lines) are overlaid on the experimental data points for the four datasets.
(DOCX)

Figure S4 **Fit to the SAXS data.** Blue dots: experimental data collected at ESRF bioSAXS beamline ID14-3, Error bars in Grey calculated from Poisson counting statistics. Green line: theoretical scattering from model with 6 linkers (28 residues each) added to the fixed domains positioned using the EM and SAXS derived envelope. The overall size and shape of the model match the experimental data well. The fit is not ideal as seen by the chi of 3.5 and the systematic deviation at 0.2 Å^-1^ which are caused by the flexibility of the protein in solution which cannot be fully accounted for in the rigid model.
(DOCX)

Figure S5 **Lack of inhibition of siRNA anti TNFR1A on the activation of respiratory epithelial cells by BC2L-C-Nter domain.** Small interfering RNA (siRNA)s directed against TNFR1A and TNFR1B (ON-TARGET plus SMART pool) were obtained from Dharmacon Inc. (Chicago, IL). siRNAs were transfected into BEAS-2B cells using Lipofectamine™ 2000 transfection reagent (Invitrogen) according to the manufacturer's instructions. Briefly, cells were seeded with 3×104 cells per well (24-well plates) in 1 mL of complete F12K (containing 10% FCS and antibiotics) 24 h prior to transfection. For transfection and per well, 20 nM siRNA were incubated for 20 min in 0.75 μL of Lipofectamine™ 2000 diluted in 50 μL of FCS and antibiotic-free F12K (Invitrogen). This lipofectamine/siRNA solution was mixed with 250 μL of FCS and antibiotic-free F12K, added to the cells and incubated for 8 h. The medium was replaced with 1 mL of complete F12K and the cells were used after 48 h. Sub-confluent BEAS-2B cells cultured in 24-well plates were incubated in 300 μL medium with BC2L-C-nt at either 0.1 µM or 0.3 µM. As negative and positive controls, cells were either not stimulated (NS) or challenged with 10 ng/mL of TNF-α (TNF). After 15 h, supernatants were collected and IL-8 concentrations were measured by ELISA. Each histogram is the mean ± sem of 3 experiments performed in triplicate.
(DOCX)

Table S1 **Concentration dependence of Rg and I_0_.** (calculated using AutoRg with variance estimated altering the data points used within the Guinier region). Merged data are obtained with the program PRIMUS by merging the low-angle region of the 1.27 mg/ml dataset (as the 0.66 and 0.31 mg/mL datasets showed variation in Rg of approximately 0.2 nm due to low signal-to-noise ratio) with the high-angle region of the 4.20 mg/mL dataset.
(DOCX)

Table S2 **Characteristics of the recombinant BC2L-C lectin and its domains.**
(DOCX)

Text S1 **Procedures for synthesis of methyl L-glycero-** ***α*** **-D-manno-heptopyranoside and allyl L-glycero-** ***α*** **-D-manno-heptopyranosyl-(1→3)-L-glycero-** ***α*** **-D-manno-heptopyranoside.**
(DOCX)
