# Supplementary figures and images for: Burkholderia cenocepacia BC2L-C Is a Super Lectin with Dual Specificity and Proinflammatory Activity
Source: PLoS Pathog. 2011 Sep 1;7(9):e1002238. doi: 10.1371/journal.ppat.1002238 (PMC3164656; doi:10.1371/journal.ppat.1002238)

***
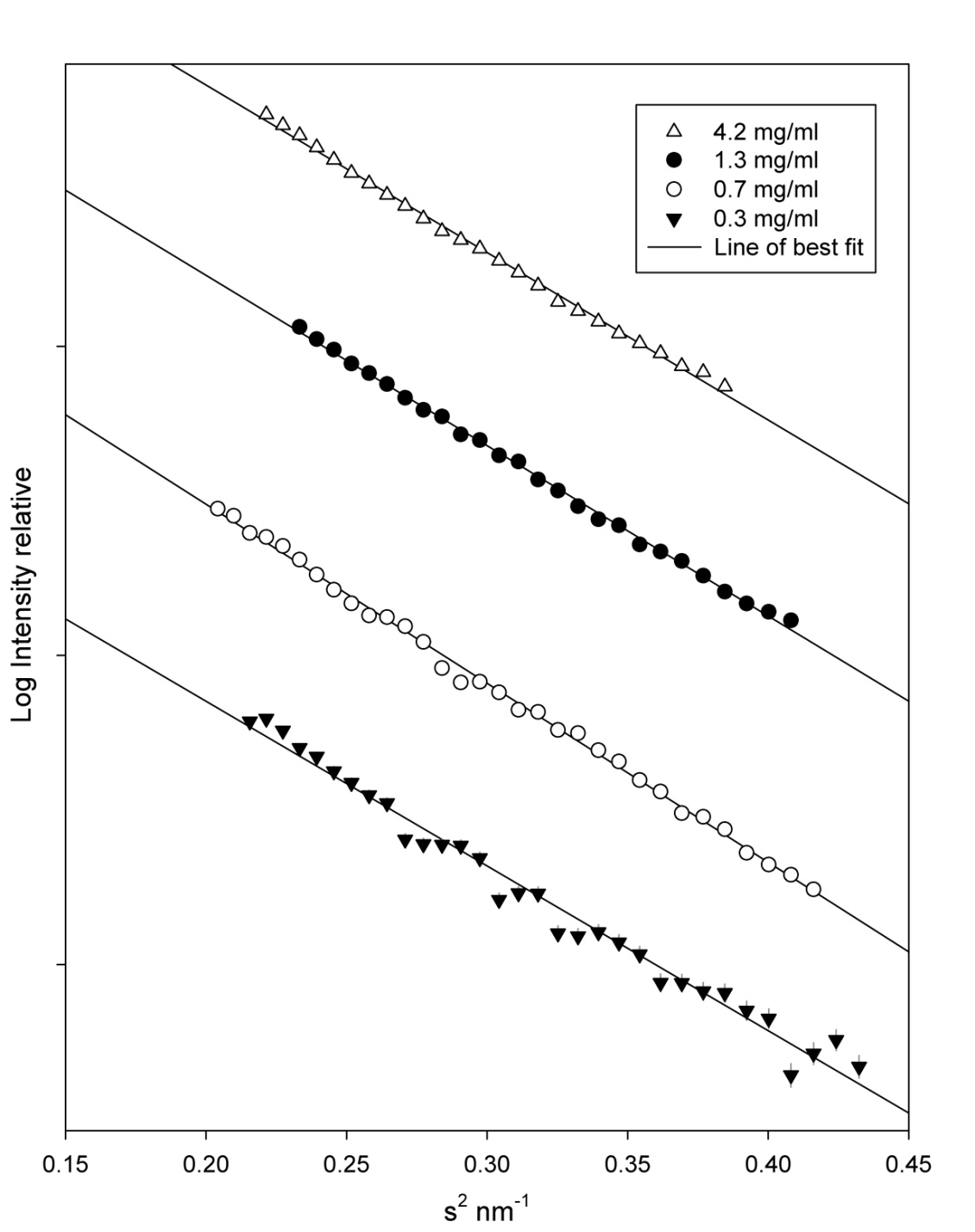
***

Supplement: Figure S3 — Guinier analysis. Calculated Guinier regions (straight lines) are overlaid on the experimental data points for the four datasets. (DOCX) [file ppat.1002238.s003.docx]

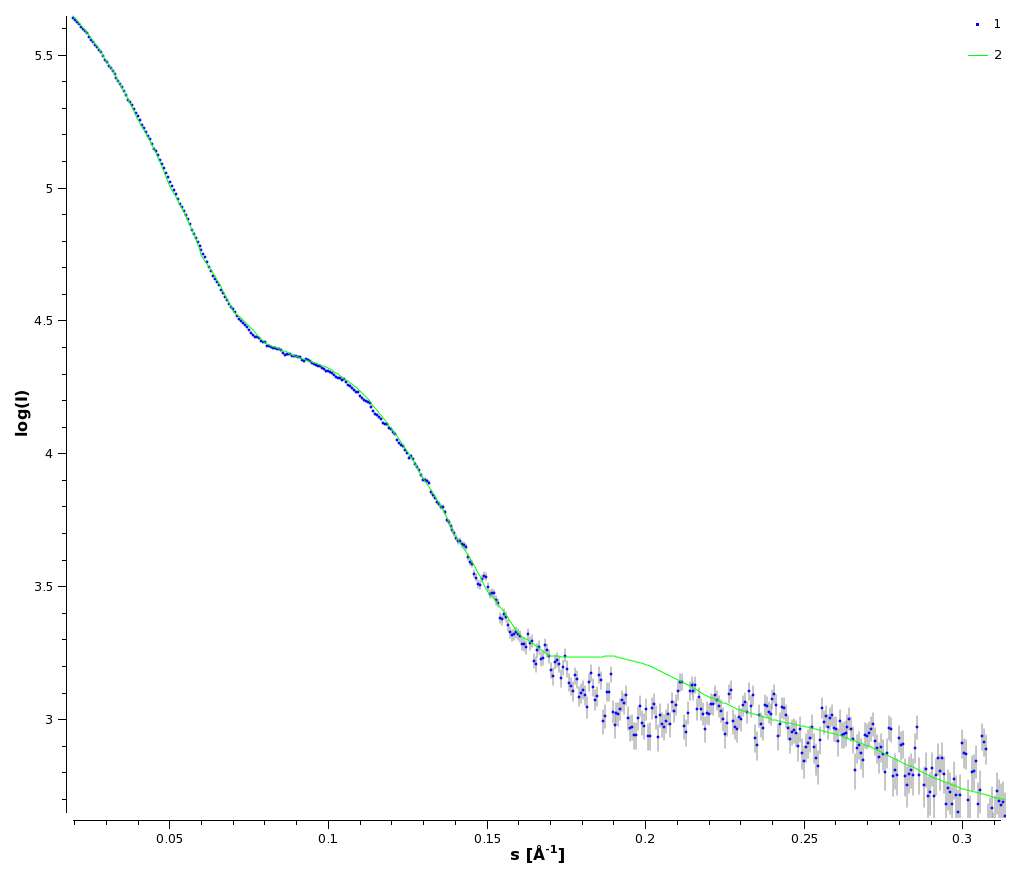

Supplement: Figure S4 — Fit to the SAXS data. Blue dots: experimental data collected at ESRF bioSAXS beamline ID14-3, Error bars in Grey calculated from Poisson counting statistics. Green line: theoretical scattering from model with 6 linkers (28 residues each) added to the fixed domains positioned using the EM and SAXS derived envelope. The overall size and shape of the model match the experimental data well. The fit is not ideal as seen by the chi of 3.5 and the systematic deviation at 0.2 Å-1 which are caused by the flexibility of the protein in solution which cannot be fully accounted for in the rigid model. (DOCX) [file ppat.1002238.s004.docx]

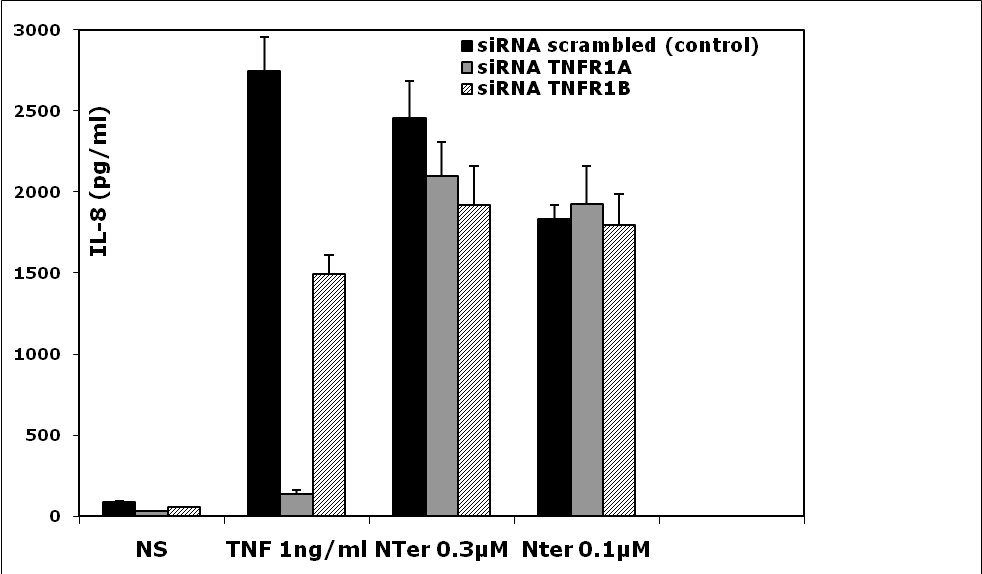

Supplement: Figure S5 — Lack of inhibition of siRNA anti TNFR1A on the activation of respiratory epithelial cells by BC2L-C-Nter domain. Small interfering RNA (siRNA)s directed against TNFR1A and TNFR1B (ON-TARGET plus SMART pool) were obtained from Dharmacon Inc. (Chicago, IL). siRNAs were transfected into BEAS-2B cells using Lipofectamine™ 2000 transfection reagent (Invitrogen) according to the manufacturer's instructions. Briefly, cells were seeded with 3×10^4^ cells per well (24-well plates) in 1 mL of complete F12K (containing 10% FCS and antibiotics) 24 h prior to transfection. For transfection and per well, 20 nM siRNA were incubated for 20 min in 0.75 μL of Lipofectamine™ 2000 diluted in 50 μL of FCS and antibiotic-free F12K (Invitrogen). This lipofectamine/siRNA solution was mixed with 250 μL of FCS and antibiotic-free F12K, added to the cells and incubated for 8 h. The medium was replaced with 1 mL of complete F12K and the cells were used after 48 h. Sub-confluent BEAS-2B cells cultured in 24-well plates were incubated in 300 μL medium with BC2L-C-nt at either 0.1 µM or 0.3 µM. As negative and positive controls, cells were either not stimulated (NS) or challenged with 10 ng/mL of TNF-α (TNF). After 15 h, supernatants were collected and IL-8 concentrations were measured by ELISA. Each histogram is the mean ± sem of 3 experiments performed in triplicate. (DOCX) [file ppat.1002238.s005.docx]
