# Supplementary material for: Burkholderia cenocepacia BC2L-C Is a Super Lectin with Dual Specificity and Proinflammatory Activity
Source: PLoS Pathog. 2011 Sep 1;7(9):e1002238. doi: 10.1371/journal.ppat.1002238 (PMC3164656; doi:10.1371/journal.ppat.1002238)
Supplement: Table S1 — Concentration dependence of Rg and I0. (calculated using AutoRg with variance estimated altering the data points used within the Guinier region). Merged data are obtained with the program PRIMUS by merging the low-angle region of the 1.27 mg/ml dataset (as the 0.66 and 0.31 mg/mL datasets showed variation in Rg of approximately 0.2 nm due to low signal-to-noise ratio) with the high-angle region of the 4.20 mg/mL dataset. (DOCX) [file ppat.1002238.s006.docx]

| **Concentration**  **(mg/ml)** | **Rg Guinier**  **(nm)** | **I_0_** | **Dmax**  **(GNOM)** |
| --- | --- | --- | --- |
| 4.20 | 5.711 ± 0.1 | 741883 | 28.42 |
| 1.27 | 5.073 ± 0.04 | 609186 | 17.76 |
| 0.66 | 4.981 ± 0.1 | 583149 | 15.59 |
| 0.31 | 4.708 ± 0.3 | 612973 | 16.48 |
| Merged data | 5.034 ± 0.04 | 606651 | 15.5 |
