# Supplementary material for: Burkholderia cenocepacia BC2L-C Is a Super Lectin with Dual Specificity and Proinflammatory Activity
Source: PLoS Pathog. 2011 Sep 1;7(9):e1002238. doi: 10.1371/journal.ppat.1002238 (PMC3164656; doi:10.1371/journal.ppat.1002238)
Supplement: Table S2 — Characteristics of the recombinant BC2L-C lectin and its domains. (DOCX) [file ppat.1002238.s007.docx]

| **Cloned Protein** | **Plasmid / Restriction Sites** | **Gene Insert Length** | **Amino Acids / Mw** | **Tag** |
| --- | --- | --- | --- | --- |
| **BC2L-C** | pRSETa / NdeI/HindIII | 813 | 271 / **28151** Da | none |
| **BC2L-C**-ct | pRSETa / NdeI/HindIII | 345 | 115 / **12 437** Da | none |
| **BC2L-C**-nt | pET25b / NdeI/HindIII | 561 | 187 (31 from Tag) / **19 257** Da | His-tag |
