# Supplementary material for: Burkholderia cenocepacia BC2L-C Is a Super Lectin with Dual Specificity and Proinflammatory Activity
Source: PLoS Pathog. 2011 Sep 1;7(9):e1002238. doi: 10.1371/journal.ppat.1002238 (PMC3164656; doi:10.1371/journal.ppat.1002238)
Supplement: Text S1 — Procedures for synthesis of methyl L-glycero- α -D-manno-heptopyranoside and allyl L-glycero- α -D-manno-heptopyranosyl-(1→3)-L-glycero- α -D-manno-heptopyranoside. (DOCX) [file ppat.1002238.s008.docx]

**Synthesis of Methyl L-*glycero*-α-D-*manno*-heptopyranoside (3).**

Methyl glycoside **3** was prepared according to literature [49]. Briefly, reducing heptose **1** was subjected to Fischer-type glycosylation with methanol followed by acetylation to furnish penta-acetate **2** in 70% yield after silica gel chromatography (Scheme). Zemplén transesterification of **2** with sodium methoxide in methanol finally produced crystalline heptoside **3** in 73% yield.

**Synthesis of Allyl L-*glycero*-α-D-*manno*-heptopyranosyl-(1→3)-L-*glycero*-α-D-*manno*-heptopyranoside (6).**

A solution of compound **4** (18 mg, 0.018 mmol, Ref. 27) in dry MeCN (2 mL) was stirred with 2% HF in MeCN (0.2 mL) for 9 h at room temperature. Solid NaHCO_3_ (0.2 g) was added and the suspension was filtered. The filtrate was concentrated and the residue was purified by chromatography on silica gel (*n*-hexane-EtOAc 1:5) to give the desilylated compound **5** (10.1 mg, 71%) as syrup. The product was dissolved in dry methanol (2 mL) and stirred with 0.1 M methanolic NaOMe (0.2 mL) at room temperature for 3 h. The solution was made neutral by adding DOWEX 50 (H+) cation exchange resin and the resin was removed by filtration. The filtrate was concentrated and finally purified on a PD-10 column (Amersham Biosciences) using water as eluant. Lyophilization afforded **6** as amorphous solid (5.4 mg, 94%). ^1^H NMR (300 MHz, D_2_O): δ = 6.02 (m, 1H, =CH-), 5.40 (dq, 1H, =CH_2_*_trans_*), 5.32 (dq, 1H, =CH_2_*_cis_*), 5.20 (br. s, 1H, H-1'), 4.85 (br s., 1H, H-1), 4.28-4.22 (m, 1H, OCH_2_), 4.13-4.03 (m, 5H, OCH_2_, H-2, H-2', H-6, H-6'), 3.98 (t, 1H, *J*_4,3_ = *J*_4,5_ 8.5 Hz, H-4), 3.96-3.89 (m, 3H, H-3, H-3', H-4'), 3.83-3.66 (m, 6H, H-7a, H-7a', H-7b, H-7b', H-5, H-5').
