# Supplementary material for: Burkholderia cenocepacia BC2L-C Is a Super Lectin with Dual Specificity and Proinflammatory Activity
Source: PLoS Pathog. 2011 Sep 1;7(9):e1002238. doi: 10.1371/journal.ppat.1002238 (PMC3164656; doi:10.1371/journal.ppat.1002238)
Supplement: Supplementary file 5 [file ppat.1002238.s001.docx]

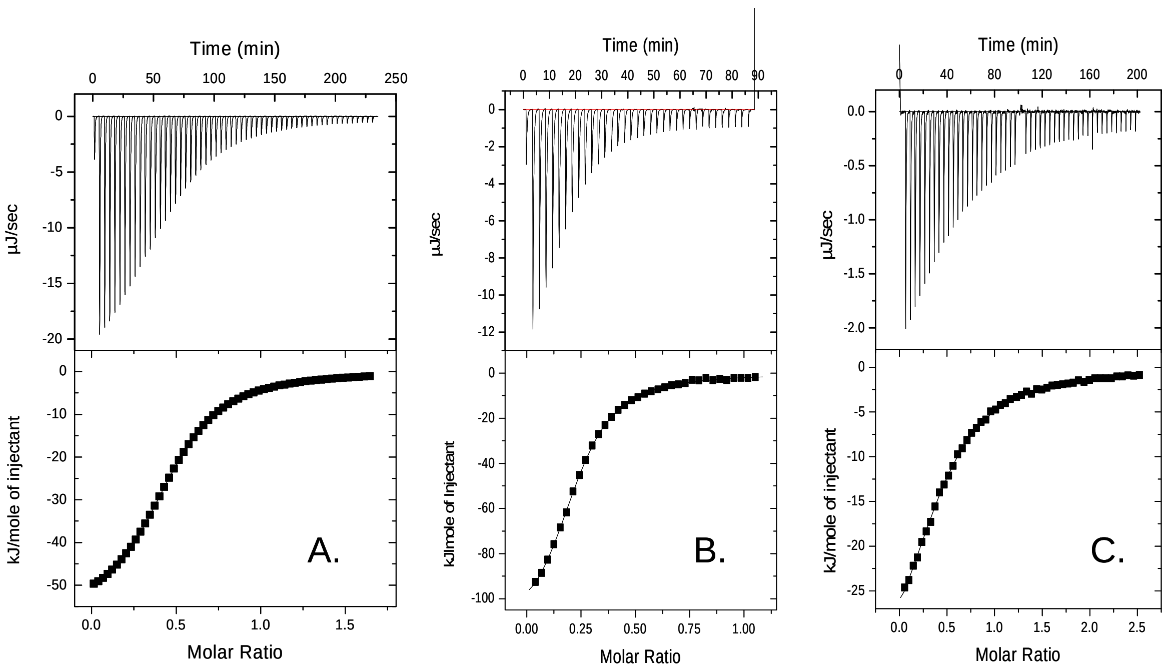
Time (min)
μJ/sec
kJ/mole of injectant
Molar Ratio
A.
Time (min)
μJ/sec
kJ/mole of Injectant
Molar Ratio
B.
Time (min)
μJ/sec
kJ/mole of injectant
Molar Ratio
C.
