# Supplementary material for: Burkholderia cenocepacia BC2L-C Is a Super Lectin with Dual Specificity and Proinflammatory Activity
Source: PLoS Pathog. 2011 Sep 1;7(9):e1002238. doi: 10.1371/journal.ppat.1002238 (PMC3164656; doi:10.1371/journal.ppat.1002238)
Supplement: Supplementary file 6 [file ppat.1002238.s002.docx]

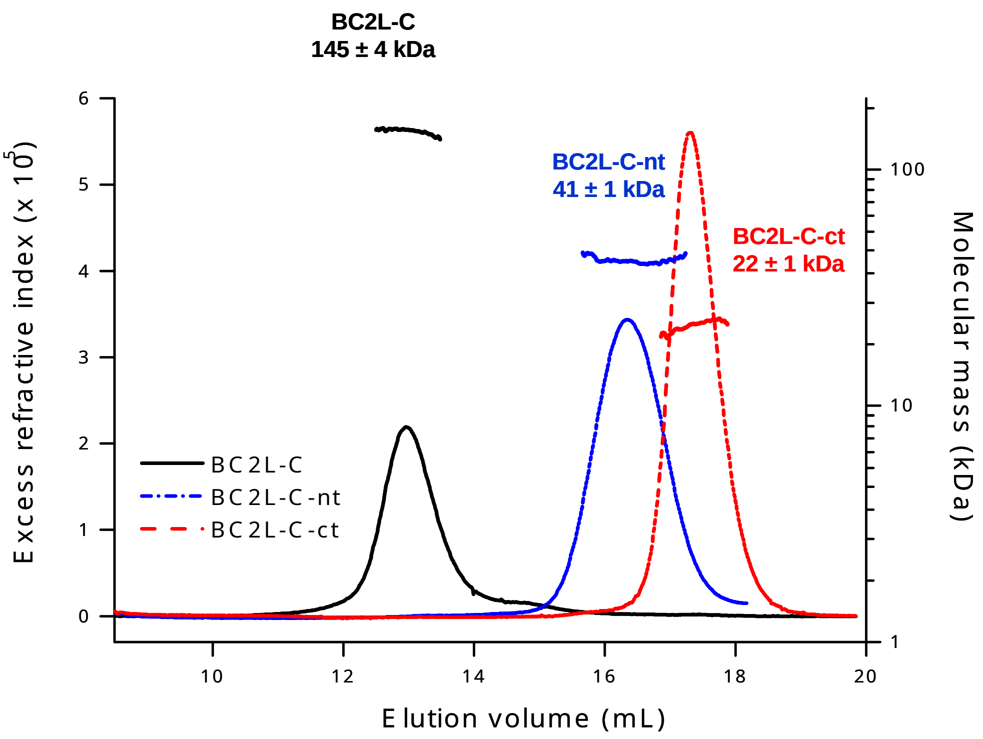
BC2L-C
145 ± 4 kDa
BC2L-C-nt
41 ± 1 kDa
BC2L-C-ct
22 ± 1 kDa
Excess refractive index (x 10⁵)
Molecular mass (kDa)
Elution volume (mL)
BC2L-C
BC2L-C-nt
BC2L-C-ct
0
1
2
3
4
5
6
10
12
14
16
18
20
1
10
100
